# Humoral Immunogenicity of SARS-CoV-2 mRNA Primary Vaccination Among People with HIV

**DOI:** 10.3390/microorganisms14040893

**Published:** 2026-04-16

**Authors:** Daniel K. Nomah, Alba G. Robles, Andreu Bruguera, Juan M. Tiraboschi, Susana Benet, Javier García-Pérez, Paloma Jimenez, Ingrid Vilaró, Gemma Navarro, Sonsoles Sánchez-Palomino, Paula Suanzes, Mercedes Garcia-Gasalla, Francisco Homar, Beatriz Mothe, Jordi Casabona, Juliana Reyes-Urueña, María J. Buzón, Jose M. Miro, The COVIHVAC Study Group

**Affiliations:** 1 Centre Estudis Epidemiològics Sobre les Infeccions de Transmissió Sexual i Sida de Catalunya (CEEISCAT), Department de Salut, Generalitat de Catalunya, 08916 Badalona, Spain; kwakyenomah@gmail.com (D.K.N.); jcasabona@iconcologia.net (J.C.); juliana.reyes81@gmail.com (J.R.-U.); 2 Infectious Diseases Department, Vall d’Hebron Institut de Recerca (VHIR), Vall d’Hebron Hospital Universitari, Vall d’Hebron Barcelona Hospital Campus, 08035 Barcelona, Spain; alba.gonzalez.robles@vhir.org (A.G.R.); paula.suanzes@vallhebron.cat (P.S.); mariajose.buzon@vhir.org (M.J.B.); 3 HIV Unit, Infectious Disease Service, Hospital Universitari de Bellvitge-IDIBELL, Universitat de Barcelona, 08907 L’Hospitalet de Llobregat, Spain; jmtiraboschi@bellvitgehospital.cat; 4 Fundació Lluita Contra les Infeccions, Infectious Diseases Department, Hospital Universitari Germans Trias i Pujol, 08916 Badalona, Spain; sbenet@lluita.org (S.B.);; 5 AIDS Immunopathogenesis Unit, Instituto de Salud Carlos III, 28029 Madrid, Spain; eoaz@isciii.es (J.G.-P.); paloma.jimenez@isciii.es (P.J.); 6 Internal Medicine Department, Hospital Universitari de Vic, 08500 Vic, Spain; ivilaro@chv.cat; 7 Unidad de Epidemiologia, Parc Tauli Hospital Universitari, Institut d’Investigaciói i Innovació Parc Taulí (I3PT-Cerca), Universitat Autònoma de Barcelona, 08208 Sabadell, Spain; gnavarro@tauli.cat; 8 HIV/AIDS Unit, Infectious Diseases Service, Hospital Clinic, Institut d’Investigacions Biomèdiques August Pi i Sunyer (IDIBAPS), University of Barcelona, 08036 Barcelona, Spain; ssanchez@recerca.clinic.cat; 9 Centro de Investigación Biomédica en Red en Enfermedades Infecciosas (CIBERINFEC), Instituto de Salud Carlos III, 28029 Madrid, Spain; mgarcia5@ssib.es; 10 Internal Medicine Department, Hospital Universitari Son Espases, Institut d’Investigació Sanitària Illes Balears (IdISBa), Universital Illes Balears, 07120 Palma, Spain; 11 Internal Medicine Department, Son Llatzer University Hospital, 07198 Palma, Spain; fhomar@hsll.es; 12 IrsiCaixa, 08916 Badalona, Spain; 13 Reial Acadèmia de Medicina de Catalunya (RAMC), 08001 Barcelona, Spain

**Keywords:** HIV, SARS-CoV-2, COVID-19, humoral response, vaccination

## Abstract

People with HIV (PWH) may exhibit altered immune responses to SARS-CoV-2 vaccination due to persistent immune dysregulation despite antiretroviral therapy. We evaluated humoral immunogenicity following mRNA SARS-CoV-2 vaccination in PWH according to CD4 T-cell count and compared responses with HIV-negative controls. The study included 57 PWH stratified by CD4 count (<200 and ≥200 cells/µL), alongside 12 HIV-negative controls. Neutralizing antibody titers (NT50) against SARS-CoV-2 pseudoviruses expressing the D614G and Omicron BA.5 spike variants were measured using a luciferase-based neutralization assay one month (M1) and six months (M6) after primary vaccination with BNT162b2 or mRNA-1273. PWH with CD4 counts ≥ 200 cells/µL demonstrated higher neutralizing titers against D614G at M1 and M6, with significant differences observed between CD4 groups (M1: *p* = 0.03; M6: *p* = 0.02). Neutralization of BA.5 was lower overall; while no overall group differences were observed at M1, higher titers were detected among individuals with CD4 ≥ 200 cells/µL at six months (*p* = 0.04). Neutralizing titers correlated positively with CD4 counts among PWH. Responses were broadly comparable between PWH and HIV-negative controls and did not differ substantially by vaccine type. These findings indicate that immune status, reflected by CD4 T-cell count, is a key determinant of SARS-CoV-2 vaccine-induced humoral responses in PWH and support prioritizing vaccination strategies for individuals with advanced immunosuppression.

## 1. Introduction

People with HIV (PWH) remain a population of clinical interest in the context of SARS-CoV-2 infection due to persistent immune dysregulation despite antiretroviral therapy (ART) [1,2]. Large population-based analyses have demonstrated that PWH may experience higher risks of severe COVID-19 outcomes, particularly those with advanced immunodeficiency or uncontrolled viral replication [3,4]. Low CD4 T-cell counts, especially <200 cells/µL, have consistently been associated with increased morbidity and mortality following SARS-CoV-2 infection [3,4].

Vaccination has played a central role in reducing the burden of severe COVID-19 globally [5]. mRNA vaccines such as BNT162b2 and mRNA-1273 showed high efficacy in randomized clinical trials conducted in the general population [6,7]. However, individuals with immunocompromising conditions, including PWH, were under-represented in these trials, leaving uncertainties regarding vaccine-induced immune responses in this population [7,8].

HIV-related immune dysregulation can affect both antibody-mediated and cellular responses to vaccination [9]. Even in virologically suppressed individuals receiving ART, immune activation, B-cell dysfunction, and incomplete immune reconstitution may reduce vaccine immunogenicity or accelerate waning antibody responses [10]. Earlier studies of SARS-CoV-2 vaccination in PWH have reported generally adequate seroconversion rates, particularly among individuals with CD4 counts ≥ 200 cells/µL [11,12]. However, several studies have also demonstrated attenuated neutralizing antibody responses among individuals with advanced immunodeficiency [13,14].

Despite these findings, evidence regarding the durability and variant-specific neutralizing activity of vaccine-induced antibodies in PWH remains limited. Many early investigations focused primarily on binding antibody responses or short-term outcomes following vaccination and did not assess functional neutralizing activity against emerging variants of concern. Additionally, longitudinal data evaluating neutralizing responses stratified by immune status in PWH remain scarce [13].

Characterizing these immune responses is particularly important given the continued evolution of SARS-CoV-2 variants with immune escape capacity. Characterizing vaccine-induced neutralizing responses against these variants in immunocompromised populations may help inform vaccination strategies and booster policies.

In this study, we evaluated the humoral immunogenicity of mRNA-based SARS-CoV-2 vaccines (BNT162b2 or mRNA-1273) in PWH receiving ART. Neutralizing antibody responses against the D614G and Omicron BA.5 variants were assessed one month and six months following primary vaccination. We further stratified responses according to CD4 T-cell counts to evaluate the impact of immune recovery and compared these responses with those observed in an HIV-negative control group.

## 2. Materials and Methods

### 2.1. Study Design

Study participants with HIV were recruited from centers participating in the PISCIS cohort, a population-based cohort that includes individuals receiving HIV care in hospitals across Catalonia and the Balearic Islands [15]. Eligible individuals were those in active follow-up, from whom participants were randomly selected.

The present analysis was primarily based on the COVIHVAC study, a prospective multicenter observational cohort that evaluated immune responses following SARS-CoV-2 vaccination among PWH. For comparison, HIV-negative control participants were included from COVIHVAX, a separate concurrent cohort study investigating vaccine-specific immune responses following two doses of mRNA-based SARS-CoV-2 vaccination [16]. Participants received a two-dose schedule of an mRNA SARS-CoV-2 vaccine (BNT162b2 or mRNA-1273). The recommended interval between doses was 21 days for BNT162b2 and 28 days for mRNA-1273, in accordance with manufacturer recommendations and national vaccination guidelines. Participants were enrolled between 22 April 2021 and 15 February 2022. Peripheral blood samples and clinical data were collected at baseline prior to vaccination (M0), approximately one month after completion of the primary vaccination schedule (M1), and six months after vaccination (M6). Baseline samples were used to confirm the absence of prior SARS-CoV-2 infection.

The primary outcome was humoral immunogenicity measured by neutralizing antibody titers against SARS-CoV-2 variants. Neutralizing responses against the ancestral D614G variant and the Omicron BA.5 variant were assessed one month and six months following vaccination. Clinical data, including comorbidities and HIV viral load, were obtained at baseline (M0). When baseline data were not available, the most recent values recorded in the PISCIS cohort database prior to vaccination were used. PWH were stratified according to baseline CD4 T-cell counts (<200 and ≥200 cells/µL).

### 2.2. Participant Eligibility

We included adults aged ≥ 18 years who received either the BNT162b2 or mRNA-1273 SARS-CoV-2 vaccine and were able to provide informed consent. Participants with evidence of previous SARS-CoV-2 infection were excluded based on a rapid antibody test performed at baseline. Participants without available baseline CD4 T-cell counts were excluded from the analysis. The HIV-negative control participants were obtained from COVIHVAX [16]. Individuals with conditions known to affect immune responses were excluded, including autoimmune diseases, chronic kidney disease, active cancer, previous organ transplantation, immunosuppressive therapy, or immunoglobulin replacement therapy. The control group was not matched for age. For PWH, clinical information on chronic comorbidities was collected, including diabetes mellitus, hypertension, current cancer, chronic kidney disease, chronic obstructive pulmonary disease (COPD), asthma, and other relevant conditions. Comparable comorbidity data were not collected for the control group; however, participants with medical conditions known to significantly affect immune responses were excluded during recruitment. Participants who reported a positive SARS-CoV-2 test or COVID-19-compatible symptoms during follow-up were excluded from analyses from that time point onward.

### 2.3. Viral Neutralization Assay

SARS-CoV-2 spike-pseudotyped viruses were generated using a vesicular stomatitis virus (VSV) backbone encoding a luciferase reporter, as previously described [17]. Briefly, 293T cells (provided by the ACTG program) were transfected with 3 μg of the plasmid pcDNA3.1-S-CoV-2Δ19-G614, a mutation associated with enhanced transmissibility compared to the ancestral Wuhan strain, or with the Omicron BA.5 spike variant. After 24 h, the cells were infected for 2 h with a replication-deficient VSV (G*VSV-ΔG-Luc) encoding the luciferase gene. Cells were then washed with warm PBS and incubated overnight in DMEM supplemented with 10% anti-VSV-G antibodies obtained from the I1 hybridoma (ATCC CRL-2700). The following day, the supernatant containing the pseudoviruses (VSV-SARS-CoV-2) was collected and titrated in Vero E6 (TMPRSS2) cells.

To perform the viral neutralization assay, plasma samples were heat-inactivated (56 °C for 1 h) and distributed through four-fold serial dilutions (1:32 to 1:131,072) in a 96-well plate. VSV-SARS-CoV-2 pseudoviruses (0.1 MOI) were then added. After incubation with plasma samples for 1 h at 37 °C, 7.5 × 10^3^ Vero E6 (TMPRSS2) cells were added to each well and incubated for 20–24 h. Viral entry was quantified based on luminescence signals measured in lysed cells using an enzyme luminescence assay (Britelite Plus kit; PerkinElmer, Waltham, MA, USA). Each plate included controls for 100% infection (VC) and background luminescence (CC). A plasma sample with a previously characterized neutralization titer was included as an internal control.

Neutralizing antibody titers (NT50) were calculated using the Reed–Muench method and expressed as the highest plasma dilution, resulting in a 50% reduction in luciferase activity compared with the VC. Samples with NT50 values < 32 were considered negative and assigned an arbitrary titer value of 16.

### 2.4. Statistical Analysis

Baseline characteristics were summarized using descriptive statistics. Continuous variables were reported as medians with interquartile ranges (IQRs), and categorical variables were presented as frequencies and percentages. NT50 were compared between groups using non-parametric tests due to the skewed distribution of antibody titers. Comparisons between more than two groups were performed using the Kruskal–Wallis test, while comparisons between two groups were conducted using the Mann–Whitney U test. Spearman rank correlation coefficients were calculated to evaluate the association between CD4 T-cell counts and neutralizing antibody titers. All statistical tests were two-sided and a *p*-value < 0.05 was considered statistically significant. Statistical analyses were performed using Stata version 14 (StataCorp, College Station, TX, USA).

## 3. Results

### 3.1. Participant Enrollment and Follow-Up

**Figure 1:**
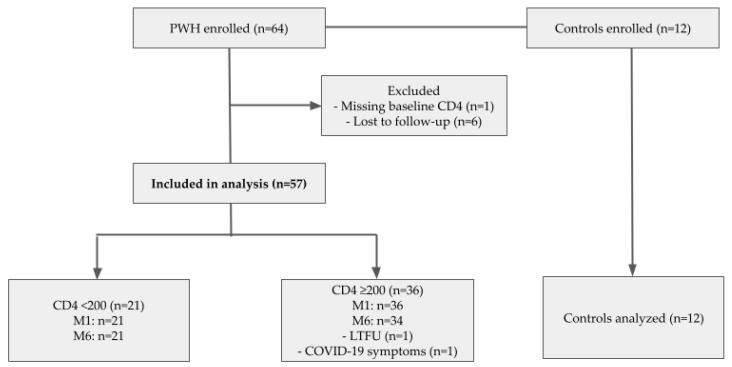
Patient flow diagram. Footnote: Abbreviations—PWH, people with HIV; CD4, CD4 T-cell count; M1, one month after completion of primary vaccination; M6, six months after vaccination; LTFU, lost to follow-up. A total of 64 PWH and 12 HIV-negative controls were enrolled. Among PWH, one participant was excluded because baseline CD4 T-cell count was unavailable and six were lost to follow-up, leaving 57 PWH in the immunogenicity analyses. At M1, samples were available for all included participants. At M6, samples were available for 21 participants in the CD4 < 200 cells/µL group and 34 participants in the CD4 ≥ 200 cells/µL group; one participant was excluded from the M6 analysis because of COVID-19 symptoms during follow-up.

Participant enrollment and follow-up in the COVIHVAC cohort are shown in Figure 1. A total of 64 PWH and 12 HIV-negative controls were initially enrolled. Among PWH, one participant was excluded due to missing baseline CD4 T-cell data, and six were lost to follow-up (LTFU), leaving 57 PWH included in the immunogenicity analyses. Participants were stratified according to baseline CD4 T-cell counts into two groups: CD4 < 200 cells/µL (n = 21) and CD4 ≥ 200 cells/µL (n = 36). At M1, samples were available for all participants in both CD4 groups. At M6, samples remained available for 21 participants in the CD4 < 200 group and 34 participants in the CD4 ≥ 200 group. Two participants in the CD4 ≥ 200 group were not included in the M6 analysis due to being LTFU (n = 1) and COVID-19 symptoms during follow-up (n = 1). All 12 HIV-negative controls completed follow-up and were included in the analyses.

### 3.2. Baseline Characteristics

Baseline characteristics of the study participants are presented in Table 1. Among the 57 PWH included in the analysis, the majority were male (98.2%), with a median age of 39.5 years (IQR 33.0–48.5). Participants in the control group were older, with a median age of 53.5 years (IQR 37.0–60.5). Most PWH were of Spanish origin (59.3%), while all participants in the control group were from Spain. The most common reported HIV transmission risk group was men who have sex with men (MSM), accounting for 77.2% of PWH, followed by heterosexual transmission (8.8%). A small proportion of participants reported injection drug use or other transmission categories. The majority of PWH had suppressed HIV viral load (93.0%), while four participants (7.0%) had detectable viral load (three of them with low-level viremia), all belonging to the CD4 < 200 cells/µL group. Median CD4 T-cell count among PWH was 404 cells/µL (IQR 156–783). Approximately 19.3% of PWH had at least one chronic comorbidity. Most participants were receiving integrase-inhibitor-based antiretroviral therapy, and the most common nucleoside reverse transcriptase inhibitor backbone was tenofovir alafenamide (TAF). Regarding vaccination, the majority of participants received the mRNA-1273 vaccine, particularly among individuals with CD4 < 200 cells/µL (90.5%).

### 3.3. Neutralizing Antibody Responses Following Vaccination

**Figure 2:**
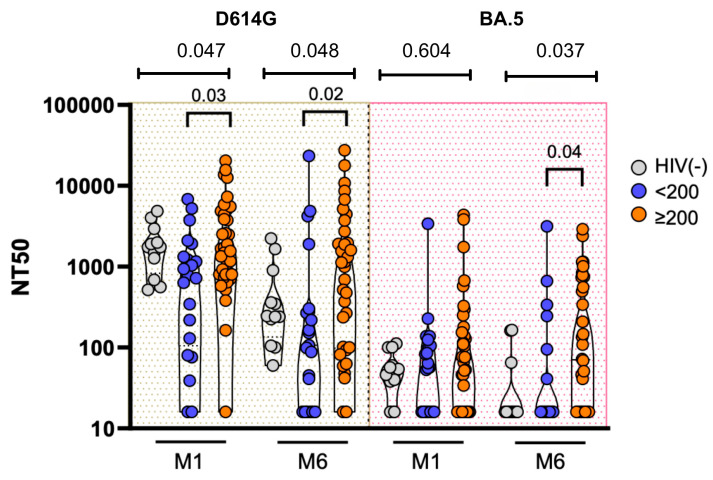
Neutralization titers (NT50) in a longitudinal cohort of people with HIV according to baseline CD4 T-cell counts. Footnote: Participants were stratified into groups with CD4 < 200 and CD4 ≥ 200 cells/µL, and HIV-negative individuals were included as controls. Neutralizing antibody titers (NT50) were measured one month (M1) and six months (M6) after completion of primary vaccination against the D614G and Omicron BA.5 SARS-CoV-2 variants using pseudotyped viruses. NT50 values are presented on a log10 scale. Differences in NT50 distributions between groups were assessed using the Kruskal–Wallis test, and the overall *p*-value for each time point is shown. Statistically significant pairwise comparisons between groups are additionally indicated.

Neutralizing antibody responses against the D614G and Omicron BA.5 SARS-CoV-2 variants are shown in Figure 2. For the D614G variant, neutralizing titers were detectable in all groups at M1. Overall differences across the three groups were observed (Kruskal–Wallis *p* = 0.047), with significantly higher titers among PWH with CD4 ≥ 200 cells/µL compared with those with CD4 < 200 cells/µL (*p* = 0.03). At M6, neutralizing titers remained detectable across groups, although titers were generally lower compared with M1. Overall differences between groups persisted (Kruskal–Wallis *p* = 0.048)**,** with significant differences between CD4 groups persisting at this time point (*p* = 0.02).

For the Omicron BA.5 variant, neutralizing titers were lower overall compared with the D614G variant. At M1, no overall differences were observed between groups (Kruskal–Wallis *p* = 0.604). However, a pairwise difference was observed between CD4 groups, with higher titers among PWH with CD4 ≥ 200 cells/µL compared with those with CD4 < 200 cells/µL (*p* = 0.04). At M6, overall differences between groups were detected (Kruskal–Wallis *p* = 0.037), with higher titers observed among PWH with CD4 ≥ 200 cells/µL compared with those with CD4 < 200 cells/µL (*p* = 0.04). When PWH were analyzed as a pooled group, neutralizing responses were comparable to those observed in HIV-negative controls at both time points for the D614G and BA.5 variants (Figure S1). Neutralizing antibody responses were also evaluated according to vaccine type among people living with HIV. NT50 titers elicited by BNT162b2 and mRNA-1273 vaccines were comparable at both M1 and M6 for the D614G and BA.5 variants (Figure S2).

### 3.4. Association Between CD4 T-Cell Counts and Neutralizing Titers

**Figure 3:**
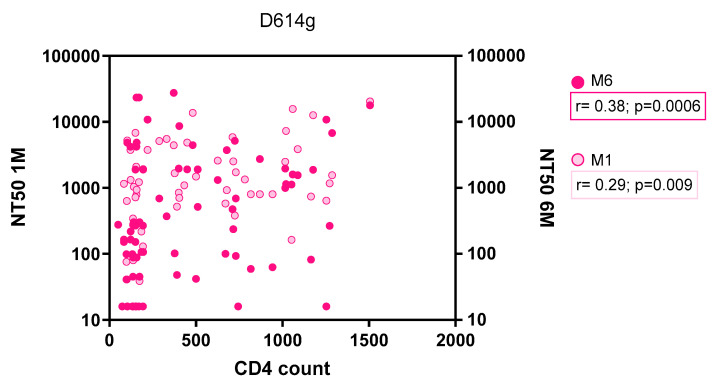
Correlation between baseline CD4 T-cell count and neutralizing antibody titers (NT50) against the SARS-CoV-2 D614G variant at one and six months after vaccination in people with HIV. Footnote: Neutralizing antibody titers (NT50) measured against the SARS-CoV-2 D614G variant were plotted against baseline CD4 T-cell counts among people living with HIV. Responses were assessed at one month (M1) and six months (M6) after completion of the primary vaccination series. NT50 values are shown on a log10 scale. Correlations were assessed using the Spearman rank correlation coefficient.

Neutralizing antibody responses were further examined in relation to baseline CD4 T-cell counts among people living with HIV (Figure 3). A positive correlation was observed between CD4 T-cell count and neutralizing antibody titers against the D614G variant at both time points. At M1, CD4 T-cell counts were modestly correlated with NT50 titers (r = 0.29, *p* = 0.009). A stronger correlation was observed at M6 (r = 0.38, *p* = 0.0006).

### 3.5. Age-Stratified Neutralizing Responses

As an exploratory analysis, neutralizing antibody responses were evaluated according to age group among people living with HIV using the cohort median age (40 years) as a cutoff (Figure S3). For the D614G variant, NT50 titers were comparable between age groups at both one month (M1) and six months (M6) following vaccination, with no statistically significant differences observed. For the BA.5 variant, lower neutralizing titers were observed among participants younger than 40 years at M1 compared with those aged ≥ 40 years (*p* = 0.04); however, this difference was not sustained at M6, where responses were similar between age groups.

### 3.6. Sensitivity Analysis

As a sensitivity analysis, multivariable linear regression models were performed among PWH to assess independent predictors of neutralizing antibody responses (Supplementary Table S1). CD4 ≥ 200 cells/µL remained independently associated with higher neutralizing titers against the D614G variant at M1 (*p* = 0.021), while detectable HIV viral load was associated with lower titers (*p* = 0.044). Age was not independently associated with neutralizing responses.

At M6, the association between CD4 group and neutralizing titers was attenuated and no longer statistically significant. For the BA.5 variant, no independent predictors of neutralizing antibody responses were identified at either time point.

## 4. Discussion

We assessed the humoral immunogenicity of mRNA-based SARS-CoV-2 vaccines (BNT162b2 and mRNA-1273) among PWH according to CD4 T-cell count and compared these responses with those observed in HIV-negative individuals over a six-month period following primary vaccination. Overall, our findings indicate that CD4 T-cell count is a key determinant of vaccine-induced neutralizing antibody responses in PWH, with individuals with CD4 counts ≥ 200 cells/μL demonstrating stronger neutralizing responses than those with CD4 counts < 200 cells/μL.

Consistent with this observation, PWH with CD4 counts ≥ 200 cells/μL exhibited significantly higher neutralizing responses against both the ancestral D614G variant and the Omicron BA.5 variant at both one month and six months after vaccination compared with those with CD4 counts < 200 cells/μL. These findings align with previous studies demonstrating that immune recovery, as reflected by higher CD4 counts, is associated with improved SARS-CoV-2 vaccine immunogenicity in PWH [14,16,18,19,20,21]. Brumme et al. [22] reported that HIV infection was associated with lower antibody concentrations and viral neutralization activity following the first vaccine dose, but these differences were largely attenuated after the second dose when adjusted for CD4 T-cell counts [22]. Similarly, a systematic review evaluating vaccine responses in PWH highlighted that lower CD4 counts were consistently associated with reduced seroconversion rates and lower antibody titers following vaccination [19]. A systematic review has also reported that PWH with CD4 counts < 200 cells/μL exhibited significantly weaker humoral and cellular immune responses one month after vaccination compared with individuals with higher CD4 counts and HIV-negative controls [23]. These findings reinforce the importance of immune reconstitution in shaping vaccine-induced responses in PWH and support focused vaccination and booster strategies for individuals with advanced immunosuppression. These findings were further supported by multivariable analyses, in which CD4 T-cell count remained independently associated with neutralizing antibody responses at one month.

Age has been proposed as an additional factor that may influence vaccine immunogenicity. In our analysis, exploratory stratified comparisons suggested some variation in neutralizing responses by age; however, these differences were not consistent across time points and were not statistically significant in multivariable analyses. Previous studies evaluating SARS-CoV-2 vaccine responses in PWH have generally focused on CD4 count and viral suppression and have rarely assessed the direct impact of age [19,20,22]. In the general population, however, older age has consistently been associated with reduced vaccine-induced immune responses due to immunosenescence [24,25]. Interestingly, the HIV-negative control group in our study did not demonstrate markedly higher neutralizing titers compared with PWH. This may partly reflect the older age distribution in the control group, which could have attenuated differences between groups. Age-related findings should be interpreted cautiously, as this analysis was exploratory and not powered to assess age as an independent predictor.

We also evaluated whether the type of mRNA vaccine administered influenced neutralizing antibody responses. Both the BNT162b2 and mRNA-1273 vaccines elicited comparable neutralizing titers at one month and six months following vaccination, regardless of the variant evaluated. These findings suggest that, among PWH, the choice between these two mRNA vaccines may not substantially affect the magnitude of the humoral response. Previous studies have similarly reported broadly comparable immunogenicity profiles between the two mRNA vaccine platforms in both the general population and immunocompromised groups [26]. These results support the overall effectiveness of mRNA vaccine technologies in generating neutralizing responses against SARS-CoV-2 variants in PWH.

Several limitations of our study should be considered. First, the HIV-negative control group was relatively small and not matched to PWH on key characteristics such as age, which may limit the robustness of comparisons. Second, although participants with evidence of prior SARS-CoV-2 infection were excluded at baseline using rapid antibody testing and clinical history, baseline neutralizing antibody measurements were not available. Therefore, the possibility of unrecognized prior infection cannot be completely excluded. Third, our cohort consisted of PWH receiving antiretroviral therapy with largely suppressed viral loads, and therefore our findings may not be generalizable to individuals with uncontrolled HIV infection or those not receiving ART. Fourth, the follow-up period was limited to six months after primary vaccination, and longer-term follow-up would be necessary to better characterize the durability of vaccine-induced immune responses in this population. Fifth, the study focused on neutralizing responses against the D614G and Omicron BA.5 variants, and responses against more recently circulating variants such as XBB, JN.1, and KP lineages were not evaluated [27]. Sixth, although multivariable regression analyses were performed, the relatively small sample size may limit the precision of estimates and the ability to detect weaker associations. Therefore, residual confounding cannot be fully excluded. Finally, the study population consisted almost exclusively of male participants, limiting the generalizability of the findings to women living with HIV.

Despite these limitations, our study provides important longitudinal data on SARS-CoV-2 vaccine immunogenicity in PWH stratified by immune status. Our findings highlight the importance of immune reconstitution and CD4 recovery in optimizing vaccine-induced protection among PWH, and support continued prioritization of vaccination strategies for individuals with advanced immunosuppression.

## 5. Conclusions

In conclusion, our study provides longitudinal evidence that the humoral immunogenicity of mRNA-based SARS-CoV-2 vaccines among people living with HIV is consistently influenced by immune status, as reflected by CD4 T-cell counts. Individuals with CD4 counts ≥ 200 cells/μL demonstrated substantially stronger neutralizing antibody responses against both the ancestral D614G variant and the Omicron BA.5 variant compared with those with more advanced immunosuppression. These findings reinforce the importance of immune reconstitution in shaping vaccine-induced responses in PWH and support prioritizing vaccination and booster strategies for individuals with low CD4 counts. Importantly, our study contributes additional real-world evidence by characterizing neutralizing antibody responses over six months after primary vaccination and by evaluating responses against both ancestral and Omicron lineage variants. We also observed broadly comparable humoral responses between the BNT162b2 and mRNA-1273 vaccines, suggesting that vaccine platform differences may play a limited role in determining neutralizing responses in this population. These findings highlight the continued need to optimize vaccination strategies for PWH with advanced immunosuppression and support ongoing monitoring of vaccine-induced immunity in this population as SARS-CoV-2 continues to evolve. Future studies should include longer follow-up periods, assessment of booster responses, and broader representation of individuals with uncontrolled HIV infection to further clarify vaccine effectiveness across the spectrum of HIV disease.

## Figures and Tables

**Table 1:** Baseline characteristics of people with HIV and HIV-negative controls.

Characteristic	All PWH (n = 57)	CD4 < 200 (n = 21)	CD4 ≥ 200 (n = 36)	Controls (n = 12)	*p*-Value *
**Gender**					>0.999
Male	56 (98.2%)	21 (100.0%)	35 (97.2%)	12 (100.0%)	
Transgender	1 (1.8%)	0	1 (2.8%)	0	
**Age, years, median (IQR)**	39.5 (33.0–48.5)	40.0 (33.0–51.0)	38.0 (33.0–45.0)	53.5 (37.0–60.5)	0.056
**Age category, years**					0.004
<40	28 (49.1%)	10 (47.6%)	18 (50.0%)	4 (33.3%)	
40–49	17 (29.8%)	4 (19.0%)	13 (36.1%)	0	
50–59	8 (14.0%)	6 (28.6%)	2 (5.6%)	5 (41.7%)	
≥60	4 (7.0%)	1 (4.8%)	3 (8.3%)	3 (25.0%)	
**Country of origin**					0.015
Spain	32 (59.3%)	12 (57.1%)	20 (60.6%)	12 (100.0%)	
Non-Spanish	22 (40.7%)	9 (42.9%)	13 (39.4%)	0	
**HIV transmission risk group**				N/A	0.907
PWID	2 (3.5%)	0	2 (5.6%)		
MSM	44 (77.2%)	18 (85.7%)	26 (72.2%)		
Heterosexual	5 (8.8%)	1 (4.8%)	4 (11.1%)		
Bisexual	3 (5.3%)	1 (4.8%)	2 (5.6%)		
Unknown	3 (5.3%)	1 (4.8%)	2 (5.6%)		
**HIV viral load**					0.015
Detectable	4 (7.0%) **	4 (19.0%)	0	N/A	
Undetectable	53 (93.0%)	17 (81.0%)	36 (100.0%)	N/A	
**Current CD4 count (cells/µL), median (IQR)**	404 (156–783)	141 (122–157)	720.5 (441–1037)	N/A	<0.0001
**At least one chronic comorbidity**					0.296
Yes	11 (19.3%)	6 (28.6%)	5 (13.9%)	N/A	
No	46 (80.7%)	15 (71.4%)	31 (86.1%)	N/A	
**Current NRTI backbone**				N/A	0.174
TAF	37 (67.3%)	13 (65.0%)	24 (68.6%)		
TDF	2 (3.6%)	1 (5.0%)	1 (2.9%)		
ABC	8 (14.5%)	4 (20.0%)	4 (11.4%)		
Other	8 (14.5%)	2 (10.0%)	6 (17.1%)		
**Current ART regimen class**				N/A	0.286
NNRTI-based	4 (7.3%)	0	4 (11.4%)		
PI-based	8 (14.5%)	3 (15.0%)	5 (14.3%)		
Integrase-inhibitor-based	43 (78.2%)	17 (85.0%)	26 (74.3%)		
**Type of vaccine received**					0.004
BNT162b2	18 (31.6%)	2 (9.5%)	16 (44.4%)	1 (8.3%)	
mRNA-1273	39 (68.4%)	19 (90.5%)	20 (55.6%)	11 (91.7%)	

Footnote: Percentages are calculated based on the number of non-missing responses. * *p*-values compare all three groups (PWH with CD4 < 200 cells/µL, PWH with CD4 ≥ 200 cells/µL, and HIV-negative controls) for variables available across groups. For HIV-specific variables where control data were not applicable, comparisons were performed between the two PWH groups only. ** Detectable plasma viral load values were 65, 216, 496, and 118,470 copies/mL. Detectable viral load was defined as ≥50 copies/mL, and undetectable viral load as <50 copies/mL. Abbreviations: PWH, people living with HIV; PWID, people who inject drugs; MSM, men who have sex with men; ART, antiretroviral therapy; NNRTI, non-nucleoside reverse transcriptase inhibitor; PI, protease inhibitor; INSTI, integrase strand transfer inhibitor; TAF, tenofovir alafenamide; TDF, tenofovir disoproxil fumarate; ABC, abacavir; IQR, interquartile range; N/A, not applicable.

## Data Availability

The study protocol and statistical code are available from the corresponding author upon request. The data for this study are available at the Centre for Epidemiological Studies of Sexually Transmitted Diseases and HIV/AIDS in Catalonia (CEEISCAT), the coordinating center of the PISCIS cohort and from each of the collaborating hospitals upon request via https://pisciscohort.org/contacte/ (accessed on 10 April 2026).

## Supplementary Materials

The following supporting information can be downloaded at: https://www.mdpi.com/article/10.3390/microorganisms14040893/s1, Figure S1: Neutralizing antibody titers in people with HIV compared with HIV-negative controls for the SARS-CoV-2 D614G and BA.5 variants at one month and six months post-vaccination. Figure S2: Neutralizing antibody titers according to vaccine type (BNT162b2 or mRNA-1273) among people with HIV. Figure S3: Neutralizing antibody responses stratified by age among people living with HIV compared with HIV-negative controls; Table S1: Multivariable linear regression analyses of neutralizing antibody titers among PWH.

## Abbreviations

The following abbreviations are used in this manuscript:

ART	Antiretroviral therapy
BA.5	Omicron BA.5 variant of SARS-CoV-2
BNT162b2	Pfizer–BioNTech mRNA COVID-19 vaccine
CD4	Cluster of differentiation 4 T lymphocyte
COPD	Chronic obstructive pulmonary disease
HIV	Human immunodeficiency virus
HIV−	People without HIV
HIV+	People with HIV
IQR	Interquartile range
M1	One month after vaccination
M6	Six months after vaccination
mRNA-1273	Moderna mRNA COVID-19 vaccine
MSM	Men who have sex with men
NT50	50% neutralizing antibody titer
PWID	People who inject drugs
PWH	People with HIV
SARS-CoV-2	Severe acute respiratory syndrome coronavirus 2
VSV	Vesicular stomatitis virus

## Appendix A

**Table A1:** COVIHVAC investigators.

Name	Affiliation
Daniel Kwakye Nomah	CEEISCAT, Badalona, Spain.
Andreu Bruguera Riera	CEEISCAT, Badalona, Spain.
Esteve Muntada	CEEISCAT, Badalona, Spain.
Juliana Reyes-Urueña	CEEISCAT, Badalona, Spain.
Sergio Moreno	CEEISCAT, Badalona, Spain.
Yesika Díaz	CEEISCAT, Badalona, Spain.
Beatriz Mothe	Fundació er Fundació Lluita contra les Infeccions, Hospital Germans Trias i Pujol, Badalona, Spain
Susana Benet	Fundació er Fundació Lluita contra les Infeccions, Hospital Germans Trias i Pujol, Badalona, Spain
Carmen Hurtado Ponce	Hospital Clínic-IDIBAPS, Barcelona, Spain
Cristina Rovira	Hospital Clínic-IDIBAPS, Barcelona, Spain
Jose Maria Miro Meda	Hospital Clínic-IDIBAPS-University of Barcelona, Barcelona, Spain
María José Maleno Martínez	Hospital Clínic-IDIBAPS, Barcelona, Spain
Núria Climent Vidal	Hospital Clínic-IDIBAPS, Barcelona, Spain
Sonsoles Sanchez-Palomino	Hospital Clínic-IDIBAPS, Barcelona, Spain
Mª del Mar Gutierrez Macia	Hospital de la Santa Creu i Sant Pau, Barcelona, Spain
Pere Domingo	Hospital de la Santa Creu i Sant Pau, Barcelona, Spain
Ingrid Vilaro López	Hospital de Vic, Barcelona, Spain
Melchor Riera Jaume	Hospital Son Espases, Palma de Mallorca, Spain.
Mercedes Garcia Gasalla	Hospital Son Espases, Palma de Mallorca, Spain.
Patricia Sorni Moreno	Hospital Son Espases, Palma de Mallorca, Spain.
Arkaitz Imaz	Hospital Universitari de Bellvitge-IDIBELL, L’Hospitalet de Llobregat, Spain
Juan Manuel Tiraboschi	Hospital Universitari de Bellvitge-IDIBELL, L’Hospitalet de Llobregat, Spain
Josep M Llibre Codina	Fundació Lluita contra les Infeccions, Hospital Germans Trias i Pujol, Badalona, Spain
Paula Suanzes Díez	Hospital Universitari Vall d’Hebron, Barcelona, Spain
Vicenç Falco	Hospital Universitari Vall d’Hebron, Barcelona, Spain
Gemma Navarro	Parc Tauli, Barcelona, Spain
Marta Navarro Vilasaro	Parc Tauli, Barcelona, Spain
Jose Alcami Pertejo	Hospital Clínic-IDIBAPS, Barcelona, Spain
