# Supplementary material for: Humoral Immunogenicity of SARS-CoV-2 mRNA Primary Vaccination Among People with HIV
Source: Microorganisms. 2026 Apr 16;14(4):893. doi: 10.3390/microorganisms14040893 (PMC13119501; doi:10.3390/microorganisms14040893)
Supplement: Supplementary file 1 [file microorganisms-14-00893-s001.zip › microorganisms-4214999-supplementary.pdf]

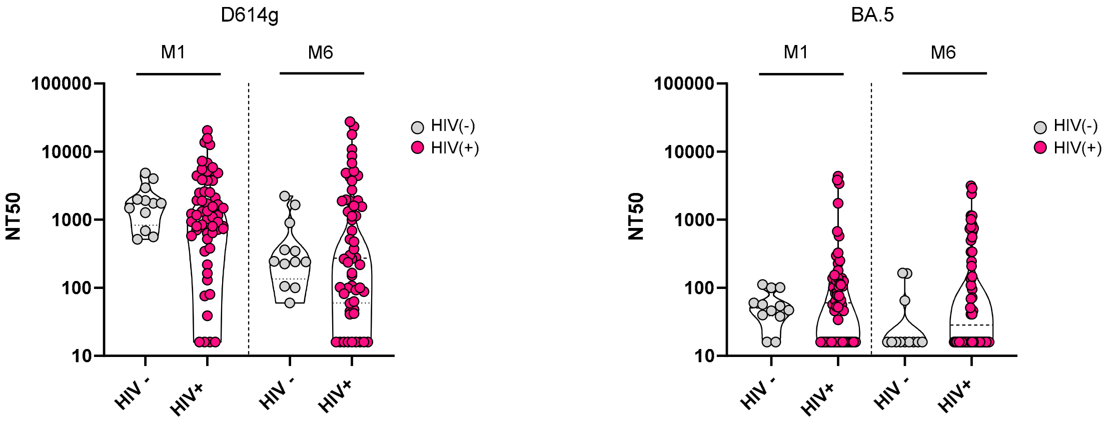

Figure S1. Neutralizing antibody titers (NT50) in people living with HIV compared with HIV-negative controls against the SARS-CoV-2 D614G and Omicron BA.5 variants at one month and six months after vaccination.

Footnote: Neutralizing antibody titers (NT50) in people living with HIV (PWH) compared with HIV-negative controls at one month (M1) and six months (M6) following completion of the primary SARS-CoV-2 vaccination series. Responses are shown for the D614G and Omicron BA.5 variants using pseudotyped virus neutralization assays. NT50 values are displayed on a log10 scale. Comparisons between groups were performed using the Mann–Whitney U test, and only statistically significant differences are indicated.

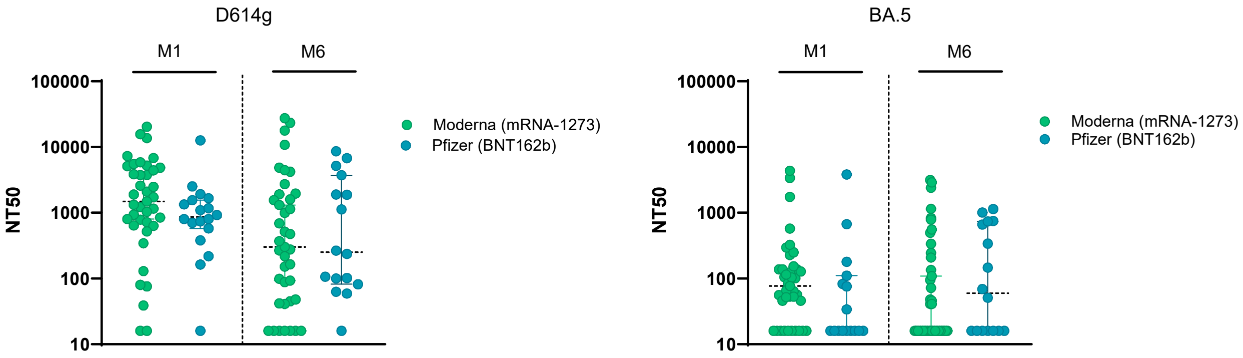

Figure S2. Neutralizing antibody titers according to vaccine type among people with HIV.

Footnote: Neutralizing antibody titers (NT50) against the SARS-CoV-2 D614G and Omicron BA.5 variants were evaluated among people living with HIV according to the mRNA vaccine received (BNT162b2 or mRNA-1273). Neutralizing responses were measured one month (M1) and six months (M6) after completion of the primary vaccination series. NT50 values are presented on a log10 scale. No statistically significant differences in neutralizing titers were observed between vaccine groups at either time point.

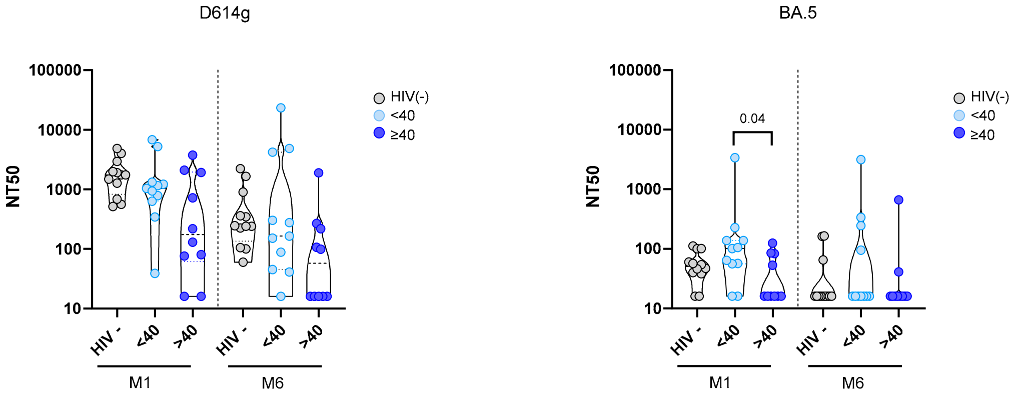

Figure S3. *Neutralizing antibody responses stratified by age among people living with HIV compared with HIV-negative controls. Footnote: Neutralizing antibody titers (NT50) against the SARS-CoV-2 D614G and Omicron BA.5 variants were evaluated according to age group among people living with HIV (PWH) and compared with HIV-negative controls. Age groups were defined using the cohort median age (<40 years vs ⩾40 years). Neutralizing responses were measured one month (M1) and six months (M6) after completion of the primary vaccination series. NT50 values are presented on a log10 scale. Differences between groups were assessed using the Kruskal–Wallis test, and statistically significant comparisons are indicated.*

Table S1. Multivariable linear regression analyses of neutralizing antibody titers among PWH

Outcome	Variable	Estimate (β)	Standard Error	p-value
**D614G – M1**	CD4 ≥200	0,52	0,22	0,021
	Age (in years)	-0,008	0,009	0,364
	Pfizer vs Moderna	-0,42	0,21	0,056
	Detectable viral load	-0,75	0,37	0,044
**D614G – M6**	CD4 ≥200	0,51	0,3	0,093
	Age (in years)	-0,017	0,012	0,17
	Pfizer vs Moderna	-0,11	0,29	0,699
	Detectable viral load	-0,63	0,5	0,214
**BA.5 – M1**	CD4 ≥200	0,21	0,22	0,343
	Age (in years)	-0,002	0,009	0,797
	Pfizer vs Moderna	-0,33	0,21	0,131
	Detectable viral load	-0,42	0,37	0,265
**BA.5 – M6**	CD4 ≥200	0,3	0,25	0,237
	Age (in years)	-0,014	0,01	0,188
	Pfizer vs Moderna	0,03	0,24	0,888
	Detectable viral load	-0,51	0,42	0,226

Footnote: Multivariable linear regression analyses assessing independent predictors of neutralizing antibody titers (NT50) among people with HIV (PWH). Outcomes are log10-transformed NT50 values for the ancestral D614G and Omicron BA.5 variants at one month (M1) and six months (M6) following primary vaccination. Models included CD4 T-cell group (<200 vs ≥200 cells/μL), age (continuous), vaccine type (Pfizer vs Moderna), and HIV viral load status (detectable vs undetectable). β coefficients represent the change in log10 NT50 associated with each variable.
